# COVID-19 and the neonatal microbiome: will the pandemic cost infants their microbes?

**DOI:** 10.1080/19490976.2021.1912562

**Published:** 2021-05-07

**Authors:** Joann Romano-Keeler, Jilei Zhang, Jun Sun

**Affiliations:** a Division of Neonatology, Department of Pediatrics, University of Illinois at Chicago, Chicago, IL, USA; b Division of Gastroenterology and Hepatology, Department of Medicine, University of Illinois at Chicago, Chicago, IL, USA; c University of Illinois Cancer Center, Chicago, IL, USA; d Jesse Brown VA Medical Center, Chicago, IL, USA

**Keywords:** COVID-19, mother-infant dyad, gut microbiome, newborn health, perinatal practice policies

## Abstract

Mortality and morbidity from SARS-CoV2 (COVID-19) infections in children remains low, including an exceedingly low rate of horizontal and vertical transmission. However, unforeseen complications to childhood health have emerged secondary to the pandemic. Few studies to date have examined unintended complications of the pandemic in newborns and infants. In this Commentary, we discuss the impact that COVID-19 may have on inheritance of the newborn microbiome and its assembly throughout the first years of life. In the early stages of the pandemic when vertical transmission of COVID-19 was poorly understood, several studies reported increased rates of C-sections in COVID-19 positive women. Initial recommendations discouraged COVID-19 positive mothers from breastfeeding and participating in skin-to-skin care, advising them to isolate during their window of infectivity. These shifts in perinatal care can adversely impact microbial colonization during the first 1000 days of life. While obstetrical and neonatal management have evolved to reflect our current knowledge of perinatal transmission, we are observing other changes in early life exposures of infants, including increased attention to hygiene, fewer social interactions, and decreased global travel, all of which are major drivers of early-life gut colonization. Composition of the gut microbiota in adults directly impacts severity of infection, suggesting a role of microbial communities in modulating immune responses to COVID-19. Conversely, the role of the intestinal microbiome in susceptibility and severity of COVID-19 in newborns and children remains unknown. The onset of adulthood diseases is related to the establishment of a healthy gut microbiome during childhood. As we continue to define COVID-19 biology, further research is necessary to understand how acquisition of the neonatal microbiome is affected by the pandemic. Furthermore, infection control measures must be balanced with strategies that promote microbial diversity to impart optimal health outcomes and potentially modulate susceptibility of children to COVID-19.

## Introduction

The paucity of SARS-CoV2 (COVID-19) related mortalities and morbidities in infants and children remains a glimmer of good news, as we witness pandemic deaths in the United States surpass 500,0000.^1^ This finding is supported by additional studies reporting exceedingly low rates of vertical transmission of COVID-19 with self-limited symptoms and self-resolution in most cases of horizontal transmission.^2^ Despite this lower prevalence and severity in children and newborns, pediatric studies are now recognizing unintended consequences of COVID–19 that significantly affect childhood health and development, including an increase in obesity, mental health disorders, myopia, and unreported child maltreatment.^3–6^ However, unforeseen repercussions of COVID-19 on newborns and infants remain largely unknown.

**Figure 1.**
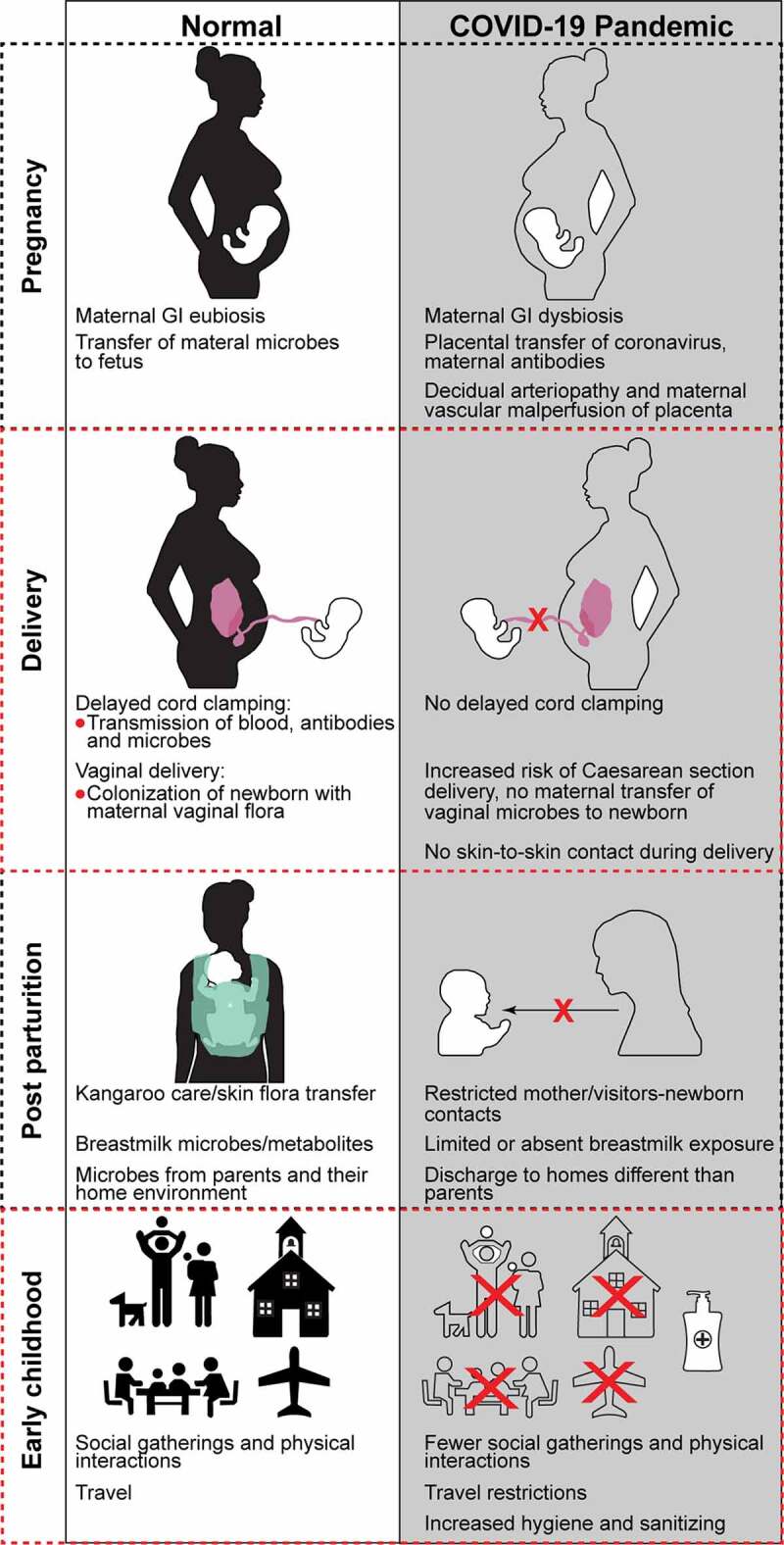
Early life microbial colonization and mechanisms by which COVID-19 may interfere with in utero and postnatal bacterial colonization

In our recent study, we observed higher rates of Cesarean sections (C-section), decreased breast milk usage, and an increased number of discharges to homes outside primary residences in twenty-one COVID-19 positive mother-infant dyads delivered at our institution.^7^ All of these outcomes have the potential to affect the developing neonatal microbiome. In addition to adaptations in perinatal care secondary to the pandemic, environmental exposures for newborns and infants have been markedly affected, including fewer social interactions, decreased enrollment in daycare, and travel restrictions. (Figure 1). These changes may have a major impact on microbial diversity. During the first 1000 days of life, the intestinal microbiome of newborns evolves to approach an adult flora by three years of age, with the first several weeks of life being characterized by highly dynamic and intricate host-microbe interactions.^8,9^ Perturbations in bacterial colonization during this window of development have been associated with chronic illnesses including inflammatory bowel diseases, cardiometabolic syndrome, and respiratory illnesses.^10^ An intestinal dysbiosis has even been correlated with severity of COVID-19 symptoms, length of hospital stay, and recovery from infection leading scientists to hypothesize that a healthy gut microbiome may be protective against COVID-19.^11,12^ As this relationship between the GI tract and COVID-19 is elucidated, it is imperative that we identify the potential impact of perinatal infection control measures and post-COVID-19 behaviors on the early life microbiome.

Fetal inheritance of maternal microbiome may occur in utero via placental exchange. However, placentas of women with severe acute respiratory distress syndrome secondary to COVID-19 have increased rates of decidual arteriopathy and maternal vascular malperfusion features which may interfere with normal bacterial exchange. In addition, an intestinal dysbiosis which is associated with COVID-19, including infected pregnant women, could impact the composition of a newborn’s pioneer bacterial communities. Microbial transfer during delivery (vaginal versus Cesarean section) and skin-to-skin care also drive bacterial colonization. Mothers who are critically ill with COVID-19 may be more likely to deliver via Cesarean section and have restricted skin-to-skin immediately post-partum or in the perinatal period. While breastfeeding and use of expressed milk are not recognized as risks for viral transmission, the rate of both may be affected during the pandemic due to maternal illness or misconceptions about breast milk safety. Mothers who are breastfeeding or pumping are instructed by medical teams on hygiene that may minimize transmission of maternal skin flora to the infant. Depending on the acuity of maternal illness or the degree to which other family members are affected, infants may be discharged to settings outside primary residences, introducing microbial communities different than those of immediate family members. Finally, temporary or more sustained adoption of pandemic-related practices, including travel restrictions, limited social gatherings, decreased physical contact with caregivers, and increased use of sanitizers, may affect the microbial diversity of the developing gut microbiome.

In this Commentary, we will explore how perinatal care models and infection control measures postnatally adopted during the pandemic to minimize horizontal and vertical COVID-19 transmission may have unintended consequences on the neonatal microbiome and childhood health outcomes. As the pandemic continues into its second year, our goal is to generate a dialogue on how to balance recommendations for newborns and infants that minimize risk of perinatal and postnatal COVID-19 infection while optimizing their microbial diversity and exposures to microbial stimuli critical for immune development and intestinal homeostasis.

## Delivery mode

Early in the pandemic, a lack of evidence on risks of transmission to the fetus and newborn contributed to increased rates of C-sections in COVID-19 positive mothers. When the low risk of vertical transmission was recognized, guidelines from trusted organizations advised against COVID-19 status alone being an indication for C–section delivery.^13–15^ However, we continue to see increased rates in infected pregnant women, in some cases attributable to deteriorating maternal status.^16^ C-sections are also higher in COVID-19 positive mothers who deliver prematurely and the relationship between COVID-19 infection and preterm birth remains unknown.^17,18^ C-section delivery is a known risk factor for early life intestinal dysbiosis due to colonization of the newborn with a mixture of potentially pathogenic organisms found on skin and in the hospital, compared with colonization by *Lactobacillus* after a vaginal delivery.^19^ Prior to the pandemic, a global rise in C-sections from 3% in 1990 to 6% in 2018 was a concern.^20^ Studies have demonstrated the significant impact of C-sections on the neonatal microbiome and an increased incidence of obesity, autoimmune disease, and atopic disorders. If the global rate of C-sections continues to accelerate, now with the added burden of COVID-19, we may observe an equivalent rise in these health conditions.

## Skin-to-skin care and rooming in

Initial recommendations from the Academy of Pediatrics (AAP) were to restrict skin-to-skin care and to separate infants from their mothers after birth to minimize COVID-19 transmission. Experiences from preterm infants, who do not receive skin to skin care (SSC) due to clinical instability and are separated from their mothers postnatally upon admission to the neonatal intensive care unit (NICU), demonstrate how interruptions in the transfer of maternal skin and oral flora can compromise newborns’ GI and immune health.^21,22^ As of August 2020, revised recommendations no longer advised against SSC or rooming in. AAP guidelines are continually reevaluated to determine the safety of such revisions, most recently in February of this year.^23^ However, limited resources at some centers may be a barrier to having dedicated nurses for COVID-19 positive mother-infant dyads in normal newborn nurseries or COVID-19 units. Finally, given the many unknowns about perinatal COVID-19 transmission, some hospitals have adopted protocols where infants remain in isolettes or other closed bed systems to reduce horizontal transmission. Discharge instructions for COVID–19 positive mothers include recommendations to limit physical contact outside of feedings. Long-term effects of limiting newborn exposure to microbial stimuli from mother’s skin and mouth during this critical first 1-2 weeks of life is still unclear.

## Breast feeding and expressed breast milk use

Breast milk is one of the most critical drivers of normal gut colonization. Earlier AAP recommendations for COVID-19 positive mothers not to breastfeed had a significant impact on the use of breast milk. As guidelines evolved with the emerging data that breast milk was an unlikely source of transmission, rates of breast milk use, including breast feeding, improved. However, many factors still limit breast milk use by COVID-19 positive mothers, including severity of maternal illness, hospital policies regarding rooming in after delivery, quarantining of mothers after discharge, and misconceptions of medical teams and parents about breastmilk’s safety. Breastfeeding and expression of milk in the immediate post-partum period is key for establishing adequate milk supply and any interruptions during this timeframe can be detrimental to the sustainability of breast milk during the first year of life.^24^ Efforts by health practitioners to counter the earlier messages about the safety of breastmilk and breastfeeding for infants of COVID-19 positive mothers should be a priority in this next phase of the pandemic. In addition, hospital resources should be directed at facilitating rooming in of COVID-19 positive mothers with their newborns as an effort to increase rates of breastfeeding at discharge.

## Home environments and hygiene measures

Environmental influences on an infant’s microbiome that have been affected by COVID-19 include exposures to households and individuals that may not normally have been in contact with newborns. For example, with the recommendation to discharge infants home with non-infected caregivers, many newborns are discharged to non-primary residences in the care of extended family members, as is the case in the cohort of patients described in our study.^7^ Household exposures are key drivers of the neonatal intestinal microbiome and by three years of age, composition of the microbiota will mirror the mother and other household members, including pets.^25,26^ Other environmental influences that infants are now encountering include a heightened attention to hygiene, such as a surge in use of antimicrobial hand sanitizers and surface disinfectants. This may drive outcomes similar to those observed with the hygiene hypothesis, including a loss in microbial diversity.^27^ It remains unknown how these early disruptions in an infant’s environment during the pandemic may impact intestinal maturation of the microbiome, immunity, and neurological development. Unfortunately, while conversations have focused on implementation of hygiene measures to prevent COVID-19 infection, few studies have explored ways to promote immune development of newborns and infants to sustain and support their relative immunity to COVID-19.

## Perinatal stress

As we shed light on the intricate communication between the gut and brain, studies suggest a relationship between maternal prenatal and postnatal stress on the composition of the infant gut microbiome.^28^ This relationship is of particular interest in pregnancies complicated by maternal COVID-19 infection where we see an increase in the number of stressors, included prolonged separations of mothers from newborns.^29^ AAP guidelines recommend COVID-19 positive mothers maintain a distance of at least six feet from their infants and they are also encouraged to wear masks when interacting with their infants, in addition to gowns and gloves, during the immediate period of infectivity. All of these recommendations can impair mother-infant bonding. In cases where infants are delivered prematurely and require admission to the NICU, COVID-19 positive mothers are not allowed to enter the NICU and may face time-consuming retesting policies after their 14-day quarantine. More research is necessary to understand gut-brain communication during perinatal stress and its impact on normal microbial exchange between mothers and their infants. In the meantime, centers implementing safety measures to minimize COVID-19 transmission should also be attentive to the effects of these measures on maternal stress. In instances where infection risks still pose barriers to mother’s visitation, technology-based interfaces, including web-based cameras, may provide a vehicle to increasing mother-infant bonding and decreasing maternal stress.

## Daycare and travel exposure

Early education and child care programs across the country have reported substantial drops in enrollment attributable to statewide or voluntary center closures throughout the pandemic along with parental preferences not to enroll in daycare in order to limit COVID-19 exposures.^30^ While longitudinal studies have suggested that entry into childcare versus continued care at home by parents does not impact an infant’s microbial composition, research in this area is nascent and data from existing studies reflect pre-COVID-19 behaviors.^31^ Restricted travel either due to state mandates or personal preferences have also occurred over the last 12 months, with a 43% reduction in air travel compared with 2019.^32^ Geography and ethnicity are critical determinants of microbial composition and health, including differences in the incidence of obesity, gastric cancer, and chronic liver diseases.^33–35^ Large-scale, collaborative international cohort studies in newborns and infants that include serial stool collection for microbiome analysis might inform us on how shifts in daycare and geographic exposures both during and after the pandemic will impact the composition and diversity of early life gut microbiota.

## Conclusions

While we remain cautiously optimistic about the impact of COVID-19 on neonatal mortality and morbidity, perinatal management during the pandemic may disrupt intestinal bacterial colonization of newborns and create other long-term complications over their lifespans. In addition, an interplay between SARS-CoV-2 and the microbiome may further complicate and disrupt intestinal maturation. Similar hospital and community-based infection control measures as described above are in place across all age groups. However, this population poses a unique challenge because of the developmental window at which their gut microbiomes are being impacted. As we begin to understand the decreased susceptibility and severity of COVID-19 observed in newborns and children, dissecting the role of the gut microbiota may provide us with further answers to this striking aspect of COVID-19. A recent study reported that homogeneity of the pharyngeal microbiota in children was associated with a lower SARS-CoV-2 positivity rate compared with older patients who had more heterogeneous bacterial communities and higher positivity rates.^36^ No studies to date have investigated whether the severe outcomes of COVID-19 in older patients could be associated with an age-dependent gut microbiome. Improving our understanding of perinatal transmission, the safety of mother-newborn care guidelines, and dynamics between viruses and bacterial communities may ensure acquisition of a normal neonatal microbiome – perhaps one of the key elements protecting newborns and children from COVID-19 infections.

As we eagerly await the availability of safe and effective COVID-19 vaccines for children, studies on how to bolster immunity in children are equally as pressing. Research in this area might reveal nutrient supplements or probiotics that could be administered enterally to infants to support their microbial diversity and strengthen immune function during the pandemic. Adult vaccination rates climb daily and we have pivoted from pandemic management to recovery. In doing so, pediatric policy makers and health advocates need to reevaluate post-COVID-19 behaviors and design recommendations around social interactions, mask wearing, and hand sanitizer use that not only minimize COVID-19 infection but also restore homeostatic host–microbe interactions in children. Finally, this pandemic provides an opportunity to reprioritize counseling of pregnant women on the importance of their diets in fostering not only their own health but the optimal development of their children’s microbiome and long-term health outcomes.
